# Probiotic–Prebiotic Co-Encapsulation in Functional Dairy Foods: Current Technologies and Prospects for Integration with Milk-Derived Bioactive Peptides

**DOI:** 10.3390/biology15191701

**Published:** 2026-09-24

**Authors:** Malika Abdulzhanova, Nurassyl Bakhytzhan, Gulina Doktyrbay, Dilyar Tuigunov, Zulfiya Kachiyeva, Nurgul Amangeldi, Aigerim Abdimadiyeva, Assima Abekova, Maka Muradashvili

**Affiliations:** 1 Department of Biotechnology, Al-Farabi Kazakh National University, 71 Al-Farabi Avenue, Almaty 050040, Kazakhstan; nurassylbakh@gmail.com (N.B.); nnurka87@mail.ru (N.A.); aiymmun2013@gmail.com (A.A.); 2 Laboratory of Food Biotechnology and Specialized Food Products, Kazakh Academy of Nutrition, 66 Klochkov Street, Almaty 050000, Kazakhstan; dilyar117@gmail.com; 3 Genome Center, Asfendiyarov Kazakh National Medical University, 94 Tole Bi Street, Almaty 050000, Kazakhstan; kachieva@gmail.com; 4 Laboratory of Immunobiotechnology, Research and Production Enterprise Antigen Ltd., 4 Azerbayev Street, Abay Village 040905, Kazakhstan; assima.abekova1@gmail.com; 5 Department of Plant Diseases Monitoring, Diagnostics and Molecular Biology, Institute of Phytopathology and Biodiversity, Batumi Shota Rustaveli State University, 26 Haidar AbashidzeStreet, Kobuleti 6200, Georgia; m.muradashvili@bsu.edu.ge

**Keywords:** probiotics, prebiotics, co-encapsulation, microencapsulation, bioactive peptides, synbiotic dairy products, functional foods

## Abstract

Co-encapsulating probiotics with prebiotic substrates (PRO–PRE) is widely proposed as a route to functional dairy products, yet the terminology and the supporting evidence are applied inconsistently. This structured narrative review examines PRO–PRE co-encapsulation in dairy matrices and, as a complementary field, the encapsulation of milk-derived bioactive peptides. Scopus, Web of Science Core Collection, and PubMed were searched for publications from 1 January 2015 to 1 September 2026. Of 663 records retrieved, 420 remained after deduplication and 330 after screening; 117 were prioritized for technological assessment. Protection is consistent but partial: encapsulated *Lacticaseibacillus rhamnosus* in ice cream declined by 0.61 log CFU/g over 50 days of frozen storage, compared with 0.97 log CFU/g for free cells. Adding a substrate is not invariably beneficial, since sweet-whey–inulin microcapsules in Greek-style yogurt and inulin in ultrafiltered white cheese conferred no advantage over the carrier alone. Outcomes depend on strain, carrier, encapsulation method, and dairy matrix, and no method proved superior on every criterion. Physical co-localization was frequently reported as synbiotic function without evidence of selective substrate utilization. No validated dairy system combining a probiotic, a confirmed prebiotic, and a characterized milk-derived peptide was identified, and clinical validation is still lacking.

## Simple Summary

Yogurts and fermented milks are often sold as sources of live bacteria that may support digestive health. Keeping enough of those bacteria alive through manufacture, storage, and the passage through the stomach is difficult. One approach is to enclose them in tiny protective capsules, sometimes together with a substrate intended to support beneficial micro-organisms in the gut. This review asks how well that actually works. Drawing on studies published since 2015, we compare the main ways of making such capsules, among them spray-drying, freeze-drying and extrusion, and we look as well at capsules made for the small protein fragments released from milk proteins. Encapsulation usually slows the loss of live bacteria, though not always, and adding the carbohydrate does not automatically help. Much depends on the bacterial strain, the capsule material, and the food itself. Human intervention evidence for complete co-encapsulated PRO–PRE systems remains limited.

## 1. Introduction

Functional foods are now designed, more and more often, as delivery systems for live micro-organisms and other biologically active components. A probiotic is a live microorganism that confers a health benefit on the host when administered in adequate amounts [1]; a prebiotic, a substrate selectively utilized by host micro-organisms that likewise confers a health benefit [2]. Both are widely used in functional food formulations, among them fermented and non-fermented dairy products [3]. What either achieves in practice, though, depends on the microorganism and the substrate, on the amount administered, on the food matrix, and on the conditions under which the product is processed and stored [1,2,3].

Keeping the number of viable probiotic cells high enough through manufacture, storage, and gastrointestinal transit remains a major technological challenge. Survival depends on the strain itself, on how the product is processed and stored, on the acidity and composition of the food matrix, and on the adverse conditions met in the gastrointestinal tract [4,5]. Microencapsulation has accordingly been examined as a way of protecting the cells and carrying them to the intestine, where they can then be released in a controlled manner [6,7]. Much depends, though, on the carrier material, on the encapsulation technique and the conditions under which it is performed, on the properties of the particles obtained, and on the food matrix for which the system is intended [6,7].

A further route to functional foods lies in combining probiotics with prebiotic substrates. The ISAPP consensus draws a distinction here between complementary and synergistic synbiotics. In a complementary synbiotic, an established probiotic and an established prebiotic act independently of one another; in a synergistic synbiotic, the substrate is designed specifically to be selectively utilized by the co-administered microorganism, and the individual components need not meet the established criteria for probiotics and prebiotics when assessed on their own [8].

Co-encapsulation brings the probiotic and the prebiotic into a single delivery system, usually one particle or carrier, which may improve their physical protection, keep them spatially associated, and allow their delivery or release to be coordinated [3,9]. Sharing a carrier, though, neither demonstrates that the substrate is selectively utilized nor establishes that the formulation confers a health benefit; both call for experimental evidence [8]. In this review, accordingly, the term “co-encapsulation” is reserved for systems in which probiotic and prebiotic are incorporated into the same particle or carrier. Where the probiotic is encapsulated and the prebiotic added separately to the food matrix, the product is described as a synbiotic formulation containing an encapsulated probiotic rather than as a confirmed co-encapsulation system. Such a formulation is classified as a synbiotic only when evidence demonstrates that the combined formulation confers a health benefit in accordance with the consensus definition [8].

Probiotic–prebiotic delivery systems have recently been studied in goat cheese, in liquid, Greek and frozen yogurts, in stirred yogurt, in fermented whey-based beverages and in ice cream [10,11,12,13,14]. Taken together, they suggest that a well-chosen carrier system can improve probiotic survival during processing and storage, and that it may also alter the physicochemical, rheological, and sensory properties of the product. Direct quantitative comparison among these studies is difficult because they differ in probiotic strain, prebiotic substrate, wall material, encapsulation method, storage period, gastrointestinal simulation protocol, and the metrics used to characterize encapsulation performance.

Examples of this configuration include Doogh formulated with alginate-encapsulated *Lactiplantibacillus plantarum* LS5 and separately added inulin, and chocolate milk formulated with whey-protein-encapsulated lactic acid bacteria and separately added konjac root powder [15,16].

Bioactive peptides originate in milk proteins and are released from them by enzymatic hydrolysis, microbial fermentation, or gastrointestinal digestion. Antioxidant, ACE-inhibitory or antihypertensive, antimicrobial, and immunomodulatory activities have all been reported for such peptides [17]. Their stability and activity can be affected by processing conditions, by interactions with the food matrix, and by digestion itself, and encapsulation has therefore been examined as a way of protecting peptide-containing fractions and modulating their release [18]. Among the delivery systems reported for milk-derived peptides and protein hydrolysates are spray-dried gelatin or chitosan microhydrogels [19], water-in-oil-in-water double emulsions [20], phosphatidylcholine nanoliposomes [21], niosomes [22], electrospun pullulan nanofibers [23], and gum Arabic microcapsules produced by spray- or freeze-drying [24]. In the literature identified here, these peptide-delivery systems have generally been studied apart from probiotic–prebiotic co-encapsulation, and their direct integration remains insufficiently explored.

For all these advances, the literature identified in this review contained almost no validated three-component systems in which a probiotic, a confirmed prebiotic, and a milk-derived bioactive peptide share a single delivery platform. Xie et al. [25] came closest, using whey-protein hydrolysates both as growth-supporting media and as protective carrier materials for *Lacticaseibacillus rhamnosus* CICC22152 during spray-drying, freeze-drying, refrigerated storage, and simulated gastrointestinal digestion. Their work offers a useful technological link between probiotic protection and the utilization of milk-protein hydrolysates. It did not, though, establish those hydrolysates as prebiotics in the sense of the consensus definition, nor did it include a separately characterized bioactive peptide cargo or a final dairy-food matrix. The study is best regarded, then, as a bridging technological model rather than a validated PRO–PRE–peptide formulation. A further caution follows from the antimicrobial activity of some milk-derived peptides [17]: their compatibility with a co-delivered probiotic calls for evaluation at strain level and cannot be assumed.

This review accordingly compares current technologies for probiotic–prebiotic co-encapsulation, sets true co-encapsulation apart from other synbiotic formulation strategies, and assesses their technological performance and compatibility with dairy matrices. Delivery systems for milk-derived bioactive peptides are examined alongside them, with a view to identifying design principles transferable to established PRO–PRE carriers. What is new here is the bringing together of two research areas that have developed separately, within one analytical framework: system architecture, component compatibility, dairy-matrix effects, technological performance, scalability, regulatory constraints, and the evidence that validated multifunctional PRO–PRE–peptide dairy products will require.

## 2. Literature Search and Review Methodology

### 2.1. Review Design and Scope

This structured narrative review deals with probiotic–prebiotic (PRO–PRE) co-encapsulation technologies and their applications in functional dairy foods. Delivery systems for milk-derived bioactive peptides were treated as a complementary technological field, with particular attention to how far they might be integrated with PRO–PRE systems. The evidence was synthesized qualitatively.

### 2.2. Literature Search Strategy

We searched Scopus, Web of Science Core Collection and PubMed, with the searches last updated on 1 September 2026. The principal publication window ran from 1 January 2015 to that date. Earlier foundational studies and consensus publications were kept where they were needed to define key concepts, to explain established encapsulation principles or to describe how the field developed technologically.

Three complementary search directions were used: probiotic–prebiotic co-encapsulation and its application in dairy products; the encapsulation and delivery of milk-derived bioactive peptides and protein hydrolysates; and systems bringing together probiotics, proposed prebiotic substrates, milk-derived peptides or hydrolysates, and encapsulation or co-delivery technologies. The core Boolean expressions were:

- (“co-encapsulation” OR coencapsulat* OR “co encapsulation” OR “co-delivery” OR “co delivery” OR microencapsulat*) AND probiotic* AND (prebiotic* OR synbiotic*) AND (dairy OR milk OR yogurt OR yoghurt OR cheese OR “ice cream” OR whey OR “fermented milk”);
- (encapsulat* OR microencapsulat* OR nanoencapsulat* OR “delivery system*” OR “controlled release”) AND (“milk-derived peptide*” OR “bioactive peptide*” OR “whey protein hydrolysate*” OR “casein hydrolysate*” OR lactoferrin OR glycomacropeptide) AND (dairy OR milk OR whey OR casein);
- probiotic* AND (prebiotic* OR synbiotic*) AND (“bioactive peptide*” OR “milk-derived peptide*” OR “whey protein hydrolysate*” OR “casein hydrolysate*” OR “casein protein hydrolysate*”) AND (encapsulat* OR coencapsulat* OR “co-delivery” OR carrier*).

The syntax was adapted to each database: TITLE-ABS-KEY fields were searched in Scopus, the Topic field in Web of Science, and Title/Abstract fields in PubMed. The main evidence base was limited to peer-reviewed original research articles and reviews published in English. Preprints, conference abstracts, corrections, and editorials were excluded, and non-peer-reviewed sources were used only for regulatory or commercial context, never as evidence of biological efficacy.

Reference lists of relevant reviews and primary studies were also screened for foundational and consensus publications that the database searches had missed. Sources identified only in this way were not counted among the database records.

### 2.3. Record Management and Selection Criteria

Search results were imported into Zotero 7 and combined into a master collection. Duplicates were identified in Zotero and verified manually. Removal of 243 duplicates from the 663 records retrieved by the database searches left 420 unique records for title and abstract screening.

A publication was retained if it addressed one or more of the following:

- co-encapsulation of probiotics and proposed prebiotic substrates within the same particle or carrier;
- encapsulated probiotics incorporated into dairy or candidate synbiotic food products;
- encapsulation or controlled delivery of milk-derived bioactive peptides or protein hydrolysates;
- effects of encapsulation on probiotic survival during processing, storage or simulated gastrointestinal digestion;
- release behavior, or effects of encapsulated components on the physicochemical, rheological or sensory properties of dairy products;
- carrier materials or processing technologies relevant to the future integration of probiotics, prebiotics and milk-derived bioactive peptides.

Studies dealing primarily with animal feed, veterinary applications, pharmaceutical or injectable delivery, cosmetics, active food packaging or meat products were excluded, as were studies of non-milk-derived peptides unless they offered information directly transferable to dairy delivery systems.

Title and abstract screening, followed where necessary by verification against accessible full texts, excluded 90 records and left 330 in the broad evidence pool.

### 2.4. Thematic Classification of the Evidence

Retained records were classified as PRO–PRE co-encapsulation systems, milk-derived peptide-delivery systems, candidate integrated systems, or indirect and transferable technological evidence. Publications dealing with definitions, biological mechanisms, regulatory requirements, and commercialization were treated as background literature.

Of the 330 records retained in the broad evidence pool, 117 were prioritized for detailed technological assessment: these addressed one of the three core review domains directly and provided sufficient methodological detail or outcome data for qualitative assessment. They comprised 95 records on reported or candidate probiotic–prebiotic encapsulation architectures, 21 on the encapsulation or delivery of milk-derived peptides, and one describing a candidate probiotic–milk-protein-hydrolysate system. The group of 117 was a technology-focused subset, not the final set of included sources. In the synthesis, records from that subset were cited only where they supported a particular statement, table entry or comparison; overlapping reports, records whose information could not be verified or extracted adequately, and records that did not contribute to the synthesis were not cited individually. The final bibliography also draws on selected reviews, consensus publications and regulatory documents from the wider evidence pool, which support the definitions and the contextual interpretation. The 108 cited sources therefore constitute the evidence base actually used, not a numerical reduction of the 117.

### 2.5. Data Extraction and Evidence Synthesis

Detailed data extraction was carried out wherever the methodological information available allowed it. Abstract-only publications were used only for qualitative statements that their abstracts support directly, and were kept out of the quantitative comparisons.

From probiotic-containing systems, the variables extracted were the microorganism and strain; the functional components and their location, inside the carrier or outside it; component concentrations; carrier composition; encapsulation method; particle size and morphology; whichever encapsulation, entrapment, immobilization or survival metric the authors reported; viable-cell counts; processing and storage conditions; survival in simulated gastrointestinal conditions; release behavior; the dairy matrix; effects on product properties; and the comparator treatments used.

For peptide-containing systems, the additional variables were the source protein; the peptide-production method; the degree of hydrolysis or molecular-mass range; peptide identity or fraction; the biological activity reported and the assay behind it; carrier composition; encapsulation efficiency; stability during processing, storage and simulated digestion; release behavior; activity retained after release; and application in model or finished food matrices.

Formulations were classified by their physical architecture and by the functional evidence available. Confirmed PRO–PRE co-encapsulation was recorded only where the probiotic and the proposed prebiotic substrate had been incorporated into the same particle or carrier. Systems in which the probiotic was encapsulated but the substrate added separately to the food matrix were classified as encapsulated probiotics with an externally added substrate, and co-encapsulation was recorded as unconfirmed wherever the available information did not settle whether both components occupied the same carrier.

Physical co-localization was not taken as sufficient evidence of synbiotic function. A formulation was classified as a confirmed synbiotic only where the evidence showed that the combination conferred a health benefit in accordance with the consensus definition, and classification as a synergistic synbiotic required evidence in addition that the co-administered microorganism utilized the substrate selectively [8]. Systems without such evidence were described by their physical architecture, or as candidate synbiotic formulations. Combinations of probiotics with plant extracts, antioxidants, vitamins or other bioactive compounds were treated as technological analogues unless the additional component satisfied an established definition of a prebiotic.

The studies were compared qualitatively on particle characteristics, viable-cell retention, storage stability, survival in simulated gastrointestinal models, release behavior, effects on dairy-product quality, scalability and suitability for dairy applications. Encapsulation efficiency, encapsulation yield, entrapment efficiency, immobilization efficiency and survival after processing were kept as distinct outcomes, their definitions and calculations differing from one study to the next. Given the heterogeneity of strains, carrier materials, processing conditions, dairy matrices, storage protocols, gastrointestinal models and analytical procedures, no statistical pooling or meta-analysis was attempted. Technological and in vitro findings were considered separately from biological effects supported by animal or human studies.

## 3. Results and Discussion

### 3.1. Mechanistic Basis of PRO–PRE Interactions

#### 3.1.1. Physicochemical Protection and Substrate-Mediated Effects

The performance of a probiotic–prebiotic delivery system depends on a number of variables that are difficult to separate in practice: the physiological characteristics of the strain, the composition of the carrier, particle size and morphology, processing conditions, mass-transfer properties, the storage environment and release behavior [3]. Encapsulation may improve stability during storage and reduce the loss of viable cells under simulated gastric and intestinal conditions [3,26], although the drying processes commonly used to produce the capsules subject the cells to dehydration and to oxidative, thermal and osmotic stress [25]. The carrier itself can be a source of difficulty at either extreme. Networks that are too restrictive may limit the transport of nutrients and the diffusion of metabolites, with a consequent reduction in cellular activity during fermentation [27]; carriers of insufficient stability may release their contents under conditions that do not correspond to the intended delivery site. Alginate–oligosaccharide beads that remained stable under gastric conditions and disintegrated in the intestine illustrate the importance of the pH-dependent behavior of the wall material [26].

Co-encapsulation is used here in the sense given in Section 2.5, with both components incorporated into the same particle or carrier. At the material level, the type and concentration of the polysaccharide or oligosaccharide used, whether inulin, fructooligosaccharides, a resistant starch or another substrate, affect the solids content of the feed, its viscosity, glass-transition behavior, network density and particle morphology [3,28,29]. Structures that are compact, or that show little porosity, may act as a barrier to environmental stressors. Morphological observation on its own, however, does not demonstrate that permeability has been reduced; this is better confirmed by rheological, thermal, permeability, or mass-transfer measurements. Compact and smooth alginate–inulin microbeads have been reported by Afzaal et al. [30], and Krunić et al. [31] found that whey-protein-hydrolysate–alginate beads formed a less porous matrix than the corresponding whey-protein-concentrate carriers.

Some sugars and oligosaccharides serve a further purpose during dehydration, acting as thermo- or cryoprotective components. Two mechanisms have been proposed for this: replacement of the water-mediated interactions around cellular membranes and proteins, and formation of an amorphous glassy matrix in which molecular mobility is reduced. Neither operates independently of the system, since carrier composition, residual moisture, storage humidity, and the glass-transition temperature of the matrix all bear on the outcome [32]. Fructooligosaccharides illustrate this. Used alone, they have a relatively low glass-transition temperature and tend to make the powder sticky during spray-drying, whereas in combination with whey-protein isolate, or with denatured whey-protein isolate, both powder formation and probiotic retention improve, provided the core-to-wall ratio is appropriate [28]. A separate spray-drying study of resistant-starch composite matrices demonstrated formulation-dependent differences in glass-transition behavior, surface integrity, thermal protection, and probiotic survival [29].

The experimental evidence therefore points to a protective contribution that is specific to the formulation and to the strain. Liao et al. [26] found that adding selected oligosaccharides to alginate beads improved both the encapsulation yield and the survival of *Limosilactobacillus fermentum* relative to alginate alone, with fructooligosaccharides performing best of the substrates examined. Raddatz et al. [33] reported longer storage stability for *Lactobacillus acidophilus* in pectin microparticles containing hi-maize, inulin, or rice bran than in pectin microparticles prepared without these ingredients.

Adding a proposed prebiotic does not, however, always give better protection. Among spray-dried sweet-whey systems, the highest viability after drying was obtained with sweet whey alone, and the sweet-whey–polydextrose formulation failed to protect the cells during heat treatment [34]. In Greek-style yogurt, sweet-whey–inulin microcapsules did not provide an evident additional protective effect, and the decline in probiotic survival during simulated gastrointestinal digestion was greater in this formulation than in the other treatments [35]. The presence of a prebiotic ingredient is therefore not in itself evidence of improved carrier performance.

A second potential mechanism operates only after release from the carrier. In a complementary synbiotic, the probiotic and the prebiotic are selected independently, and each meets the established definition of its category, whereas in a synergistic synbiotic the substrate is designed specifically for selective utilization by the co-administered microorganism [8]. Improved survival during processing, storage, or simulated digestion therefore demonstrates neither selective substrate utilization nor a synbiotic health benefit.

More direct mechanistic evidence comes from Huang et al., who showed that the human milk oligosaccharide 6′-sialyllactose promoted the growth and acetate production of *Bifidobacterium animalis* F1–7, and that the combination increased short-chain fatty-acid production in an in vitro fecal fermentation system [36]. These findings are nonetheless specific to the microorganism, the substrate and the experimental model used. They cannot be generalized to PRO–PRE carriers as a class, nor read as evidence of a health benefit in vivo.

The available evidence thus supports two conceptually separate contributions of a prebiotic-containing carrier: a physicochemical one during processing, storage and gastrointestinal transit, and a possible substrate-mediated one after release. Viability, carrier structure, storage stability and release experiments are sufficient to assess the first. The second calls for evidence that host micro-organisms utilize the substrate selectively, and, where a synergistic synbiotic is claimed, that the co-administered microorganism does so. Classification of the combined formulation as a synbiotic requires, in either case, evidence that it confers a health benefit.

The physicochemical protection provided by the carrier and the potential substrate-mediated effects following release are summarized schematically in Figure 1.

#### 3.1.2. Prospective Interactions in Integrated PRO–PRE–Peptide Systems

Extending a PRO–PRE carrier to include milk-derived bioactive peptides introduces potential interactions that remain largely untested in integrated systems. The sequence- and matrix-dependent stability, localization, and gastrointestinal release of encapsulated milk-derived peptides are examined in detail in Section 3.4 [19,20,37].

Xie et al. [25] offer a useful technological bridge between probiotic protection and milk-protein hydrolysates. They evaluated highly and moderately hydrolyzed whey proteins as combined culture media and drying matrices for *Lacticaseibacillus rhamnosus* CICC22152, and found that the hydrolysate-based media increased biomass production and supported probiotic survival through drying, storage, and simulated gastrointestinal digestion. A second candidate bridging system comes from Krunić et al., whose cell-release measurements led them to infer a less porous matrix in whey-protein-hydrolysate–alginate beads than in the corresponding whey-protein-concentrate–alginate beads; the protein–alginate carriers also maintained high probiotic survival under simulated gastrointestinal conditions [31]. Together, these findings suggest that milk-protein hydrolysates may perform both nutritional and structural functions in probiotic carriers.

Antagonistic interactions must be considered to be well. Pei et al. [38] isolated two antimicrobial peptides, RVMFKWA and KVISMI, from yak-milk protein hydrolysates; both inhibited several bacterial species, and KVISMI inhibited selected fungi in addition. Since the peptides were not tested against probiotic strains, or within an integrated delivery system, the study offers no direct evidence of antagonism in a PRO–PRE peptide carrier. It does, however, give a biological reason to check each peptide fraction against the intended probiotic strain, at the concentrations expected inside the carrier and after release, before a co-encapsulated formulation is developed.

Stability and compatibility are not the only concerns: bitter or hydrophobic peptide fractions may also spoil the sensory acceptability of the finished dairy product [39]. Controlled spatial separation, or sequential release, might keep a probiotic and an antimicrobial peptide from direct contact during processing and storage while still allowing each component to be released at the intended stage of gastrointestinal transit. That remains a design hypothesis and needs direct experimental validation.

Future integrated systems should therefore document carrier architecture, probiotic viability, peptide identity and the activity retained, release behavior and compatibility with the dairy matrix. Where synbiotic function is claimed, selective substrate utilization and a health benefit must be demonstrated as well [8].

### 3.2. Conventional PRO–PRE Encapsulation Technologies

The technologies covered in this section differ in their operating conditions and in the stage at which the delivery structure is formed. Spray-drying and spray cooling form the particles during atomization; extrusion and emulsification generate droplets that are hardened afterwards, usually by gelation; freeze-drying is primarily a dehydration step, applied either to cell–carrier mixtures or to preformed particles. Gelation is therefore treated here as a particle-hardening mechanism rather than as a wholly independent way of generating particles [7,40].

The systems in this section are classified throughout by the operational criteria set out in Section 2.5. Only formulations in which the probiotic and the prebiotic substrate occupy the same carrier are described as PRO–PRE co-encapsulation systems.

#### 3.2.1. Spray-Drying

In spray-drying, the probiotic cells and the prebiotic substrate are dispersed in a liquid carrier and atomized into a stream of heated air. The solvent evaporates rapidly and leaves dry particles in which the components are held within a protein, carbohydrate, or composite matrix. Particle formation and cell survival depend on the composition of the feed, its total-solids content and viscosity, the atomization conditions, the inlet and outlet temperatures, the flow of drying air, the residence time and the residual moisture of the powder. Milk proteins, whey ingredients, maltodextrin, gum Arabic, alginate, inulin and other carbohydrates have all been used as carrier materials, alone or in combination [7,40].

The method’s advantages are continuous operation, comparatively high throughput, low unit cost and a powder that can be transported, dosed and incorporated into dry or reconstituted dairy products. Its main limitation is that atomization and rapid dehydration expose the cells to thermal, oxidative, osmotic and mechanical stress at the same time. A short residence time in the drying chamber is therefore no guarantee of probiotic survival, and protection must be demonstrated for the particular strain, carrier composition and processing conditions [7,40].

Experimental work illustrates how far this depends on the formulation. Alvarado-Reveles et al. [41] spray-dried *Lacticaseibacillus rhamnosus* GG with agave fructans in a buttermilk-protein matrix; compared with particles prepared without fructans, the combined formulation survived 14 days of storage at −20 °C and simulated gastrointestinal conditions better, and the encapsulated cells still grew in and acidified goat milk. In another dairy-derived system, the best retention of *Bifidobacterium animalis* subsp. *lactis* BB-12 during spray-drying and subsequent storage was obtained with full-fat goat milk alone and with full-fat goat milk containing inulin. The oligofructose formulation gave no comparable protection, and surface cracks in the particles were associated with lower viability [42]. Adding a prebiotic ingredient, in other words, does not compensate for unsuitable matrix properties or poorly controlled drying.

Spray-drying remains one of the most industrially applicable ways of producing PRO–PRE powders, particularly where a free-flowing ingredient is wanted. Its performance, though, should be judged from the viable cells retained immediately after drying and across the intended storage period, together with particle properties and release behavior, and not from powder yield or nominal encapsulation efficiency [7,40].

#### 3.2.2. Freeze-Drying

Freeze-drying begins with the freezing of a cell–carrier dispersion, or of a preformed particle system, after which the ice is removed by sublimation under reduced pressure. Doing without a heated air stream reduces direct thermal exposure, but it does not remove the damage caused by freezing and dehydration. How the process performs depends on the strain, the composition of the carrier, the freezing conditions, the residual moisture, and the storage environment afterwards [7,40].

Freeze-drying as a means of preserving cells needs to be distinguished from freeze-drying as part of an encapsulation process. Drying cells together with a soluble protective material does not necessarily give a discrete carrier. Under the operational criteria set out in Section 2.5, a preparation can be classified as PRO–PRE co-encapsulation only if particle formation has been demonstrated and both functional components are shown to be retained within the particle. Particles formed by extrusion, emulsification, or gelation and freeze-dried afterwards are a different case: there the process is a combined encapsulation and dehydration.

Wang et al. [43] prepared inulin–alginate beads containing *Lactiplantibacillus plantarum* and coated them with skim milk before freeze-drying. The coated inulin–alginate formulation improved cell survival during drying and kept viability higher than the less complex comparator formulations over seven weeks of storage and on exposure to simulated gastric fluid and bile salts. Gedik and Karahan incorporated *Lactiplantibacillus plantarum* AB6-25 and *Saccharomyces boulardii* T8-3C separately into alginate-based particles containing demineralized whey powder and lactobionic acid. After freeze-drying, both encapsulated micro-organisms survived simulated gastrointestinal conditions better than free cells, and refrigerated particles held viable counts above the authors’ stated target level for 180 days [44]. What was evaluated there was a carrier system, not a finished dairy food. Since neither selective utilization of lactobionic acid nor a health benefit of the combination was demonstrated, the system is treated here as a candidate PRO–PRE architecture and not as a validated synbiotic.

Freeze-drying suits heat-sensitive formulations and can give high recovery of viable cells when the formulation and the process conditions are chosen well. Against this stand its industrial limitations: batch operation, long processing times, high energy demand, the need for vacuum equipment, and powders that are sensitive to moisture and oxygen in storage. It is, therefore, not universally superior to spray-drying, and the choice between them must take account of storage stability, rehydration behavior, production scale and performance in the intended dairy matrix [7,40].

#### 3.2.3. Extrusion

In extrusion-based PRO–PRE co-encapsulation, a hydrocolloid feed containing the probiotic cells and a proposed prebiotic substrate is passed through a nozzle into a hardening bath. Sodium alginate is the usual gelling polymer and calcium chloride the usual crosslinking solution, though alginate is often combined with proteins, starches, gums or other polysaccharides. Bead diameter and size distribution depend on the nozzle diameter, the viscosity of the feed, the flow rate, surface tension and the distance between the nozzle and the hardening solution [7,45].

Conventional extrusion is normally carried out without high-temperature drying, so immediate thermal injury to the cells can be kept to a minimum, and the method is straightforward enough at laboratory scale. What limits it is the size of the beads: simple dripping systems give relatively large and sometimes heterogeneous particles, throughput is low, the nozzle can block, and the particles must be collected, washed and, where required, dried. In smooth dairy products, large beads may be perceptible and may spoil the mouthfeel. Scaling the method up therefore means controlling droplet generation and reducing particle size, not simply multiplying laboratory-scale nozzles [7,45].

Haghshenas et al. [46] used simple extrusion to prepare alginate–psyllium beads containing the candidate probiotic *Enterococcus durans* 39C together with inulin or with fenugreek-containing material proposed to have prebiotic activity. They reported encapsulation efficiencies above 98%, survival above 47% under simulated digestive conditions, and retention of viable cells through 28 days of yogurt storage at 4 °C, with the encapsulated cells surviving these conditions better than free cells. Technological protection is clear from these data, but they establish neither the probiotic status of the strain nor selective utilization of the substrates, and they show no health benefit of the combination.

Kavas et al. [10] provide more direct evidence from a dairy matrix. Using extrusion and 1% sodium alginate, they prepared microcapsules containing *Lacticaseibacillus paracasei* and *Bifidobacterium longum*, either without a proposed prebiotic or with fructooligosaccharides or inulin, incorporated them into white goat cheese, and followed the cheeses through 180 days of storage at 4 °C. Viable counts held up better in the cheeses containing prebiotic-enriched microcapsules than in the probiotic-only formulations [10]. The study thus documents a same-carrier PRO–PRE architecture in a dairy product, though better viability by itself demonstrates neither selective substrate utilization nor a synbiotic health benefit.

Extrusion offers mild processing conditions and can yield PRO–PRE carriers that are suitable for incorporation into dairy products. Whether it is practical depends on reaching an acceptable particle size and sensory profile while keeping droplet formation reproducible and throughput adequate at industrial scale.

#### 3.2.4. Emulsification

In emulsification-based encapsulation, an aqueous phase carrying the probiotic cells and the carrier polymers is dispersed in a food-grade oil phase. Where a same-carrier PRO–PRE system is intended, the proposed prebiotic substrate goes into the aqueous phase before the droplets form. The droplets are then hardened by internal or external ionic gelation. Internal gelation relies on the gradual release of calcium ions from a poorly soluble calcium salt included in the droplets themselves; external gelation relies on the introduction of a soluble calcium source, usually calcium chloride, into the emulsion. Particle size and size distribution follow from the viscosity of the dispersed phase, the oil-to-water ratio, the emulsifier concentration, and the homogenization conditions. Set against conventional gravity-driven extrusion, emulsification can give smaller particles and form many beads at once [4,47].

Frakolaki et al. [48] encapsulated *Bifidobacterium animalis* subsp. *lactis* BB-12 by emulsification followed by external gelation, using a range of alginate-based carrier formulations. Best overall protection over 30 days of refrigerated and frozen storage, and after exposure to simulated gastric and intestinal conditions, came from the formulation in which milk replaced water and which contained 1% alginate, 5% glucose, and 2% inulin. Since BB-12 and inulin were incorporated into the same beads, the formulation satisfies the architectural criterion for PRO–PRE co-encapsulation given in Section 2.5. The particles were nonetheless evaluated as an encapsulated ingredient and not within a finished dairy product, so how they perform in an actual dairy matrix is still an open question.

Emulsification works under relatively mild thermal conditions and gives smaller particles than simple extrusion, but it calls for an oil phase and an emulsifier, and the particles then must be recovered and washed, and in some applications dried. Residual oil or surfactant, downstream separation, particle yield, and any effects on flavor and texture accordingly need to be checked before the particles go into a dairy product. The trade-off is better control of particle formation against downstream processing that is more involved than in conventional extrusion [4,48].

#### 3.2.5. Ionic Gelation and Prospective Enzymatic Gelation

Probiotic carriers are hardened ionically by one of two routes, external or internal gelation. In external gelation, calcium ions diffuse into the alginate-containing droplets from the surrounding hardening solution. In internal gelation, a poorly soluble calcium salt is included in the droplets, and the calcium ions are released from it afterwards, usually by acidification. Internal gelation can distribute the crosslinking ions more gradually, though what it achieves depends on the rate of calcium release, the pH, the polymer concentration and the rate of gelation [7,47].

Silva et al. [49] prepared alginate–gelatin microgels containing *Lactobacillus acidophilus*, with and without fructooligosaccharides, by external gelation. The fructooligosaccharides gave a more interconnected carrier network, and both formulations improved probiotic survival over free cells during yogurt storage and simulated gastrointestinal exposure. By the architectural criteria adopted in this review, the alginate–gelatin–fructooligosaccharide formulation is a case of same-carrier PRO–PRE co-encapsulation.

Cezarino et al. [11] later incorporated these probiotic and PRO–PRE microgels into liquid, Greek-style, and frozen yogurts, where their performance differed from one matrix to another. Probiotic viability during storage was highest in liquid yogurt, whereas protection during simulated digestion was greater in frozen yogurt. The microgels left the apparent viscosity of the liquid and frozen products unchanged but lowered it in Greek-style yogurt. Results obtained for isolated particles, then, cannot simply be carried over to every dairy matrix.

Tarifa et al. [50] used ionotropic gelation to prepare pectin and pectin–inulin microgels containing *Lacticaseibacillus casei* ATCC 393 or *Lacticaseibacillus rhamnosus* GG. Mean particle diameters were 56–118 μm, and the encapsulation efficiency was about 96%. Encapsulated cells stayed above 7 log CFU through 42 days of storage at 4 ± 1 °C, while resistance to simulated gastrointestinal digestion varied with the strain, the incubation time, and whether commercial or Jerusalem artichoke inulin was used [50]. The protection given by ionically gelled PRO–PRE carriers is thus dependent on both strain and formulation.

Enzymatic crosslinking is a related route for protein- and peptide-based carriers, and a much less developed one. Nourbakhsh et al. [51] treated whey-protein hydrolysates with transglutaminase before micro-emulsification and acid-induced gelation, obtaining peptide-based nanoparticles that carried gallic acid. Enzyme-assisted gel formation from dairy-derived protein hydrolysates is technologically feasible based on this evidence. The system contained neither a probiotic nor a confirmed prebiotic, however, so it is transferable evidence for future PRO–PRE–peptide carriers rather than an established PRO–PRE co-encapsulation system.

Ionic gelation forms particles under mild conditions and works with food-grade polysaccharides. How well it performs depends on the uniformity of crosslinking, the pH of the environment, competing ions, swelling, and the retention of soluble substrates. Evaluation in the intended dairy product remains necessary, since milk composition, acidity, storage conditions, and product structure may all change carrier stability, probiotic survival, release behavior, and sensory properties.

#### 3.2.6. Coacervation

Coacervation rests on the separation of an initially homogeneous polymer solution into a polymer-rich coacervate phase and a polymer-poor equilibrium phase. Simple coacervation involves one polymer and can be induced by changing temperature, pH, ionic strength, or solvent conditions. Complex coacervation usually arises from electrostatic association between oppositely charged biopolymers, most often a protein and a polysaccharide. The polymer-rich phase deposits around the dispersed core and is then stabilized by cooling, ionic gelation, crosslinking, or drying [7,45].

The process can be run under comparatively mild thermal conditions and may give relatively dense carrier structures. It is, however, highly sensitive to the polymer ratio, molecular charge density, pH, ionic strength, mixing conditions, and the composition of the continuous phase. Minerals, proteins and organic acids in dairy products may accordingly change both phase separation and carrier stability compared with what is seen in simplified laboratory media. Agglomeration, incomplete deposition of the coacervate phase, particle recovery and reproducibility on scale-up are further technological concerns [7,45].

Complex coacervation has been used in several studies to combine probiotics with functional components that are not prebiotics. Eratte et al. [52] co-encapsulated *Lacticaseibacillus casei* and tuna oil in whey-protein-isolate–gum-Arabic complex coacervates, which were then turned into powders by spray or freeze-drying, and followed probiotic viability and fermentation activity along with the oxidative stability of the oil over 90 days of storage. Useful, as this is for probiotic–lipid compatibility and for the use of dairy proteins in coacervate walls, it is not PRO–PRE co-encapsulation, since tuna oil is not a prebiotic substrate.

Juárez-Trujillo et al. [53] examined an architecture closer to the point. *Bifidobacterium animalis* subsp. *lactis* and agave fructans of high degree of polymerization were first incorporated into a double emulsion; the system was then coacervated with sodium alginate and dried by freeze-drying, spray-drying, or convective drying. Convective drying gave the highest reported encapsulation efficiency and the best survival after simulated gastrointestinal exposure, while the spray- and freeze-dried preparations kept more viability during storage at room temperature. As the microorganism and the fructans were incorporated into the same delivery structure, the preparation satisfies the architectural criterion for PRO–PRE co-encapsulation, although neither selective substrate utilization nor a health benefit of the combination was demonstrated.

That system also brings out an interpretive limitation. Emulsification, coacervation, and drying were applied in sequence, so the protection obtained cannot be attributed to coacervation on its own, and the particles were not evaluated in a finished dairy product. Direct evidence for complex-coacervate PRO–PRE systems tested in dairy matrices is therefore still limited, and no technological superiority over alginate gelation or spray-drying has been established.

#### 3.2.7. Spray Cooling and Hybrid Approaches

Spray cooling, or spray chilling, atomizes a suspension or emulsion of the active components in a molten lipid carrier into a chamber held below the solidification temperature of that carrier. The particles form by solidification of the lipid rather than by evaporation of a solvent, so hot-air dehydration is avoided, though contact with the molten carrier can still impose thermal stress on the cells. A hydrophobic lipid matrix may limit direct contact between the incorporated cells and aqueous environments, but particle aggregation, poor wettability, incomplete release, and possible waxy sensory effects all must be evaluated for the food application in view [6,54].

Okuro et al. [55] incorporated *Lactobacillus acidophilus* together with inulin or polydextrose into solid-lipid microparticles produced by spray chilling. The process caused no significant immediate loss of viability, and the particles improved survival on exposure to simulated gastric and intestinal fluids. Over 120 days of storage, viability varied with storage temperature, storage atmosphere, and carrier composition, with the polydextrose formulation showing the most favorable overall stability.

Spray-freeze-drying is a different process. Here, the feed is atomized into a cryogenic environment, the droplets freeze rapidly, and the water is then removed by sublimation. Atomization-based particle formation is thus combined with low-temperature dehydration, but atomization, freezing, and sublimation follow one another, and processing stress is not eliminated.

Yoha et al. [56] compared spray-drying with spray-freeze-drying for *Lactiplantibacillus plantarum* NCIM 2083 in formulations containing fructooligosaccharides, whey protein, and maltodextrin. For the selected FOS: whey-protein:maltodextrin formulation, encapsulation efficiency was 89.21% after spray-drying and 96.16% after spray-freeze-drying. The spray-freeze-dried preparations survived simulated gastrointestinal digestion better, while the spray-dried ones retained viability better over 60 days of storage. This direct comparison shows that which method performs best depends on the outcome being measured: the process giving greater initial recovery or digestive protection need not give the greatest storage stability.

Spray cooling and spray-freeze-drying reduce different kinds of processing exposure and are not, therefore, universally superior alternatives to conventional drying. What makes either suitable is the strain, the carrier composition, the particle properties required, the storage conditions and the dairy application intended. Electrohydrodynamic atomization is dealt with separately, in the section on emerging technologies.

#### 3.2.8. Comparative Assessment and Methodological Limitations

Table 1 makes it clear that no encapsulation method is optimal on every criterion. Spray-drying is well suited to continuous, comparatively inexpensive, high-throughput powder production. The alternatives reduce particular forms of processing stress but bring constraints of their own: freeze-drying needs long processing times and a high energy input, conventional extrusion often gives relatively large particles at limited throughput, and emulsification, gelation and coacervation may require further particle-recovery and stabilization steps. In dairy applications, particle size, hydration behavior and effects on texture and mouthfeel constrain the choice further. Requirements of this kind partly explain the continuing interest in smaller-particle systems and in carriers based on milk or whey proteins [6,7].

Comparisons between studies still call for caution. In the literature included here, the terms “encapsulation efficiency”, “encapsulation yield”, “immobilization efficiency”, “freeze-drying efficiency” and “survival rate” were not used consistently. The value reported was, depending on the study, the proportion of cells recovered from disrupted particles, the proportion still viable after processing, or some other study-specific ratio. Particle-disruption procedures, enumeration methods, and calculation denominators differed as well. Percentages of similar magnitude are therefore not necessarily equivalent, and Table 1 keeps the metric used by the original authors and, where the information allows, identifies its calculation basis, rather than presenting all reported percentages as one directly comparable outcome.

A high reported encapsulation efficiency does not, in itself, predict performance during storage or gastrointestinal exposure. Laina et al. [57] compared freeze-drying with electrospraying of *Lacticaseibacillus rhamnosus* GG in whey-protein-isolate–inulin matrices that were then incorporated into yogurt. Freeze-drying gave the slightly higher encapsulation efficiency, while electrospraying produced denser structures and better protection during storage and simulated gastrointestinal exposure. Encapsulation efficiency, particle structure, viable-cell retention during storage, and gastrointestinal survival are thus distinct performance outcomes. The technological details of electrospraying are considered separately in the section on emerging technologies.

A second limitation lies in the heterogeneity of the simulated gastrointestinal models. The INFOGEST protocol offers a harmonized framework for static food-digestion experiments, with defined oral, gastric and intestinal stages and standardized conditions for pH, enzymes, bile, dilution and exposure time [58]. Its use across the studies examined here was not consistent. The models reported differed in gastric pH, exposure time, enzyme activity, bile concentration, food-to-fluid ratio, the sequence of the digestive stages and the point at which viable cells were enumerated. Survival values obtained with different models are accordingly indicative rather than directly comparable. Preservation of viable counts through an in vitro digestion experiment also demonstrates neither intestinal colonization nor a health benefit, and in vivo validation of complete PRO–PRE delivery systems was uncommon in the evidence set reviewed here.

Strain specificity is another important source of variation. De Andrade et al. [59] spray-dried *Lactiplantibacillus plantarum* CCMA 0359 and *Levilactobacillus brevis* CCMA 1284 in whey, whey–inulin and whey–maltodextrin matrices. Encapsulation efficiencies above 86% were reported for both strains, but their viability over 90 days of storage differed. Whey and whey–maltodextrin supported the storage stability of *Lactiplantibacillus plantarum* well; for *Levilactobacillus brevis* only whey gave a comparable result, and inulin did not improve viability under the conditions evaluated. A carrier formulation that performs well for one strain cannot, therefore, be assumed to protect another equally.

The choice of method should accordingly be matched to the probiotic strain, the proposed prebiotic substrate, the intended dairy matrix, the particle size required, the dominant processing and storage stresses, the release conditions, the production scale and the format of the final product. The comparisons made in the dairy-application sections that follow are interpreted on this basis, in relation to the particular formulation, matrix, experimental model and outcome measured.

**Table 1:** Comparative assessment of conventional, hybrid, and emerging approaches used to prepare probiotic–prebiotic carriers and related probiotic delivery systems.

Method and Technological Role	Resulting Structure or Representative Size	Representative Reported Metric(s) *	Effect on Probiotic Viability	Scalability and Cost	Dairy Applicability and Evidence Status	References
Spray-drying	Micrometer-scale dry particles, usually spherical, wrinkled, or partly collapsed; size and morphology depend on feed composition and drying conditions.	Pinto et al. [34]: yield 95.43% (sweet whey alone, non-prebiotic comparator). de Andrade et al. [59]: EE > 86% (whey, whey–inulin, whey–maltodextrin). Dantas et al. [60]: yield 88.01% (lactose-free milk–inulin). Metrics are not interchangeable.	Short residence time, but simultaneous thermal, oxidative, osmotic, and dehydration stresses. Survival is strain- and carrier-dependent; adding a proposed prebiotic does not invariably improve protection.	Industrially established; continuous, high-throughput operation; comparatively low unit cost.	Well suited to powdered and reconstituted dairy ingredients; milk, buttermilk and whey carriers are compositionally compatible, though hydration, storage stability and sensory effects remain formulation-specific.	[7,34,41,59,60]
Freeze-drying (lyophilization)	No intrinsic particle size: gives a porous cake, or dries preformed structures; 3–10 μm particles reported in one combined encapsulation–freeze-drying system.	Gedik and Karahan reported values of 88.46–99.13% across their encapsulation- and freeze-drying-efficiency measurements. These are distinct, study-specific measures rather than a method-wide EE range.	Avoids heated drying air, but freezing, solute concentration, ice crystals, osmotic and dehydration stresses remain; the outcome depends on strain, freezing conditions, carrier, residual moisture and storage.	Industrially available but batchwise, slow and energy-intensive; usually more costly than spray-drying.	Suitable for starter and probiotic powders and for drying preformed particles; rehydration, hygroscopicity, oxygen exposure and storage temperature need control.	[7,43,44]
Extrusion followed by external ionic gelation	Beads from submillimeter to several millimeters; size set by nozzle diameter, viscosity, flow, surface tension and hardening conditions.	Haghshenas et al. [46] reported EE > 98% for *Enterococcus durans* 39C in all tested alginate–psyllium formulations containing inulin or fenugreek.	Mild processing usually preserves high immediate viability and can improve survival in storage and digestion; dense gels may restrict diffusion or delay release.	Simple and inexpensive at laboratory scale, though simple dripping has low throughput and can suffer nozzle blockage; multinozzle or vibration-assisted systems improve the prospects for scale-up.	Moderately suited to smooth dairy foods, since large beads may be perceptible in yogurt or alter mouthfeel; bead size and release profile should be matched to the product.	[7,46,61]
Emulsification followed by internal or external ionic gelation	Usually smaller particles than simple dripping, but size depends on phase ratio, viscosity, mixing, emulsifier, and gelation kinetics; no defensible universal range was identified.	No method-wide efficiency range. Frakolaki et al. [48] assessed viable-cell retention and found milk-alginate-glucose-inulin beads to give the strongest overall protection among their formulations.	Mild thermal conditions; performance depends on droplet formation, carrier composition, gelation, and recovery operations.	Mixing scales readily, but separation, washing, removal of residual oil, and any subsequent drying add complexity, water and energy use, and cost.	Potentially suitable where all oils, emulsifiers and crosslinkers are food-grade; residual oil, flavor compatibility, yield and texture must be assessed in the intended dairy matrix.	[7,9,48]
Complex coacervation	Polymer-rich coatings or microcapsules; their dimensions depend on polymer ratio, charge density, pH, ionic strength, mixing and stabilization, and no reliable universal size range applies.	No method-wide PRO–PRE efficiency range can be supported, and no direct study of a confirmed dairy PRO–PRE complex-coacervation system was identified in the retained evidence set.	Potentially mild, but cells may be affected by pH adjustment, ionic strength, crosslinking, and particle-recovery steps.	Highly formulation-sensitive: reproducible control of pH, charge ratio, mixing, agglomeration, and particle recovery complicates scale-up.	Protein–polysaccharide pairs may be compositionally compatible with dairy ingredients, but the retained evidence set contains no direct evidence for confirmed dairy PRO–PRE systems, so any claim of superiority remains provisional.	[7,9,45]
Spray cooling/spray chilling	Solid-lipid microparticles, formed by atomizing a molten lipid dispersion into a cooled chamber; the structure depends on lipid crystallization and on atomization.	Okuro et al. [55] reported no marked immediate viability loss and monitored stability for 120 days; the outcomes were viable counts and survival, not a conventional EE value.	Avoids hot-air dehydration, and the lipid phase may limit fluid penetration; the molten carrier can still impose thermal stress, and performance depends on lipid composition and storage.	Potentially continuous and scalable, but temperature control, lipid crystallization, aggregation, wettability, and powder handling require optimization.	Potential for lipid-rich or frozen dairy products. Uncontrolled release, poor wettability, and a waxy mouthfeel are possible constraints.	[45,55]
Spray-freeze-drying (hybrid)	Porous dry microparticles or agglomerates; the initial droplet size is set by atomization and the final structure by freezing and sublimation.	For a fructooligosaccharide-whey-protein-maltodextrin formulation, EE was 96.16% after spray-freeze-drying versus 89.21% after spray-drying.	In the direct comparison, spray-freeze-drying gave better survival during simulated digestion, whereas spray-drying retained viability better during 60-day storage.	Multistage, time- and energy-intensive; higher cost and lower industrial maturity than conventional spray-drying.	Useful for heat-sensitive powdered ingredients, though cost, low throughput, and the handling of porous powders may limit its use in routine dairy manufacture.	[7,56]
Electrohydrodynamic atomization (electrospraying)	Tunable micro- or submicrometer particles; size depends on voltage, flow rate, nozzle geometry, conductivity, surface tension, and viscosity.	In ice cream after 90 days, viable counts were 7.37 and 7.82 log CFU/g for inulin- and resistant-starch-containing microcapsules, respectively; these are storage counts, not EE.	A low bulk temperature avoids severe thermal exposure, though electrical, interfacial, shear, and dehydration stresses remain possible; multilayer carriers can improve survival in storage and digestion.	Precise but commonly low-throughput. Multinozzle equipment and continuous collection are required for industrial scale-up.	Direct ice-cream and yogurt examples exist, and small particles may lessen the sensory impact, but the method is still emerging for industrial dairy use.	[7,57,62]
Electrospinning	Continuous fibers rather than particles; fiber diameter must not be reported as particle size.	Loading efficiency, viable recovery, and survival are study-specific and cannot be pooled with particle-based EE; no direct integrated PRO–PRE dairy electrospinning study was identified in the retained evidence set.	Low bulk temperature, but solvent exposure, osmotic effects, electrical stress, and dehydration may affect cell survival.	Generally low throughput; collector design, uniformity and continuous production remain scale-up constraints.	Potential for edible films, fibrous inserts or rapidly dissolving structures; applicability to conventional smooth dairy products is not yet established.	[7,9]

* Reported values are representative results from the cited studies rather than universal performance ranges for the corresponding methods. Original terms such as encapsulation efficiency, encapsulation yield, freeze-drying efficiency, survival rate and viable count were retained because calculation denominators, particle-disruption procedures and microbiological enumeration methods differed among studies. Values should not be compared directly unless equivalent procedures were used. Primary studies provide the formulation-specific numerical or application evidence shown in the table. Review articles are used only for general descriptions of method principles, advantages, limitations, and scale-up considerations; they are not treated as sources of the primary numerical results. Ionic gelation is not treated as a fully independent particle-generation method because it is generally used to harden droplets produced by extrusion, emulsification, or electrohydrodynamic atomization. Evidence for enzymatic gelation in confirmed dairy PRO–PRE co-encapsulation systems was insufficient for a separate quantitative comparison.

### 3.3. Emerging and Next-Generation Technologies

Emerging encapsulation approaches differ widely in technological maturity, so the evidence below is grouped into direct dairy-food applications, non-dairy-food proof-of-concept studies, and transferable engineering or biomedical findings.

The electrohydrodynamic techniques are electrospraying, which produces particles, and electrospinning, which produces continuous fibers. Both can be run at relatively low bulk temperatures, although cell survival still depends on feed viscosity and conductivity, applied voltage, flow rate, formulation composition, and collection conditions [7,63]. Gómez-Mascaraque et al. [63] encapsulated *Lactiplantibacillus plantarum* in a whey-protein-concentrate matrix by electrospraying. Once the process had been optimized, viability losses were below 1 log CFU and the powders contained more than 8.5 log CFU/g; their stability during storage and simulated gastrointestinal digestion was then evaluated. The carrier was dairy-derived, but the particles were not evaluated in a finished dairy product.

Zaeim et al. [62] provide more direct dairy evidence. They incorporated *Lactiplantibacillus plantarum* together with inulin or resistant starch into alginate–chitosan microcapsules produced by electrohydrodynamic atomization and added these to ice cream; after 90 days, viable counts were 7.37 ± 0.12 log CFU/g for the inulin particles and 7.82 ± 0.39 log CFU/g for those containing resistant starch. The study confirms a PRO–PRE co-encapsulation architecture in a dairy product, though it demonstrates neither selective substrate utilization nor a health benefit. Of the emerging methods considered here, electrospraying thus has the strongest direct dairy evidence, but industrial implementation will require higher throughput, continuous particle collection and reproducible multinozzle operation. The retained evidence set contains no primary study of an integrated dairy PRO–PRE system produced by electrospinning, which is therefore best regarded as a prospective fiber-forming platform rather than an established dairy encapsulation method.

Microfluidic techniques give precise control over droplet formation and particle-size distribution, but their direct relevance to dairy PRO–PRE systems is still insufficiently demonstrated [64]. Zhang et al. [65] built an integrated microfluidic chip with 80 droplet generators and reported a maximum production rate of 10 mL/h for single-cell-loaded alginate microgels [65]. The system was developed for biomedical cell applications rather than for probiotic or food delivery, so it illustrates an engineering route to microfluidic scale-up, not the industrial feasibility of a dairy PRO–PRE product. No validated microfluidic PRO–PRE system in a dairy matrix was identified.

Single-cell encapsulation applies a protective coating directly to individual cells. Sbehat et al. [66] prepared three-layer coatings around *Lacticaseibacillus rhamnosus* from black-seed protein together with inulin, alginate, or glucomannan. After sequential exposure to simulated gastric and intestinal fluids, viability fell by about 1.5 log for the inulin-containing coating against about 6.7 log for uncoated cells, and the coated cells were also incorporated into pectin films. This is food-oriented proof of concept rather than validation in a dairy product or evidence of a synbiotic health benefit. Production throughput, coating reproducibility, long-term storage stability, and the regulatory status of the coating materials all remain to be established.

Three-dimensional printing is a food-structuring process rather than an encapsulation method, since the cells are already protected when they enter the printable matrix. In a non-dairy flour model, extrusion-based printing caused no significant loss of *Lactiplantibacillus plantarum* viability, though survival afterwards depended on the preceding encapsulation and on post-printing treatment; the best formulation retained 79% viability after static in vitro digestion [67]. Feasibility is established as proof of concept, but applicability to industrial dairy production is not.

Technological readiness therefore varies. Electrohydrodynamic atomization has been evaluated directly in a dairy product; single-cell coating and 3D printing have mainly food-oriented proof-of-concept support outside dairy matrices; microfluidics and electrospinning rest largely on transferable laboratory or engineering evidence. None of these approaches is yet an established industrial alternative to spray-drying, freeze-drying, extrusion or gelation. Taking them further calls for evidence on production throughput, process reproducibility, food-grade carrier safety, storage and shelf-life stability, sensory compatibility, performance in real dairy matrices and economic feasibility. Where synbiotic functionality is claimed, selective substrate utilization and a health benefit must be demonstrated as well.

### 3.4. Encapsulation of Milk-Derived Bioactive Peptides: Technological Performance and Relevance to Integrated PRO–PRE Systems

The origin and reported activities of milk-derived peptides were introduced above. What this section asks is narrower: whether encapsulation preserves peptide identity and activity, whether it modifies gastrointestinal release, and whether the resulting carriers can be incorporated into dairy matrices. The distinction matters, because a reported encapsulation-efficiency value establishes neither the retention of a particular bioactive sequence, nor improved bioaccessibility or systemic bioavailability, nor a health benefit. Protein hydrolysates are complex peptide mixtures, and processing, fermentation, carrier interactions and digestion may all alter their peptide profiles and the activities measured [19,37,68,69].

Drying-based encapsulation has been studied chiefly as a way of producing stable peptide-containing powders, though the properties of the powder and the peptides retained depend on the feed composition, the carrier-to-core ratio, the drying conditions, the residual moisture and the physicochemical properties of the hydrolysate [68,70]. Corrêa et al. [24] compared spray-drying with freeze-drying of a sheep-whey hydrolysate carried in gum Arabic. In that formulation, spray-drying gave the better powder, with 3.9% moisture and 18.9% hygroscopicity, and showed 65.7% ABTS-radical-scavenging activity, 78.4% iron-chelating activity and 70.1% ACE inhibition; the simulated gastrointestinal procedure the authors used did not substantially affect these activities. Freeze-drying cannot, on this comparison, be assumed to protect peptide preparations better than spray-drying, since the ranking depends on the hydrolysate, the carrier, the process conditions and the outcome measured. Antioxidant or ACE-inhibitory activity shown in vitro should likewise not be taken as evidence of an antihypertensive or other biological effect in vivo.

The principal pathway from milk proteins to encapsulated peptide preparations is summarized schematically in Figure 2.

Work on microhydrogels shows that peptide protection can be sequence-specific. Gómez-Mascaraque et al. [19] incorporated a whey-protein hydrolysate into spray-dried gelatin or chitosan microhydrogels and followed peptide stability through yogurt fermentation and simulated gastrointestinal digestion. Of the 21 peptides identified as susceptible to fermentation, chitosan microhydrogels protected five, and gelatin protected only one of those five. Chitosan encapsulation left the peptide profile recovered after digestion largely unchanged, but so small a number of protected sequences shows that encapsulating a hydrolysate does not protect all its constituent peptides equally [19]. This sequence dependence agrees with studies of whey-peptide nanoliposomes, in which peptide molecular weight, charge, compositional homogeneity and hydrophobicity affected peptide localization and peptide–carrier interactions even where overall encapsulation efficiency changed little [71,72]. Carrier performance is therefore best evaluated, wherever possible, at the level of individual sequences or defined peptide fractions, and not through total protein or peptide measurements alone.

Digestion of the carrier can itself change the composition and the apparent activity of the released fraction. Alvarado et al. [69] encapsulated a <3 kDa ACE-inhibitory whey-peptide fraction in alginate–collagen, alginate–gum-Arabic, and alginate–gelatin matrices, with alginate–gum Arabic giving the highest reported encapsulation efficiency at about 95%. After simulated digestion, the released fraction showed 85% ACE inhibition against 74% before digestion, but digestion also released sequences that had not been identified in the starting fraction [69]. Other work on milk proteins confirms that gastrointestinal digestion is an active transformation stage and not simply a release test. Giroux et al. [20] showed the release of casein-derived peptides from double emulsions to depend on peptide hydrolysis, oil-phase composition and intestinal lipolysis, and in a bovine-lactoferrin system Xing et al. [73] found that carrier composition and the successive gastric and intestinal stages altered the diversity and persistence of the fragments detected. The rise in bulk ACE-inhibitory activity reported by Alvarado et al. [69] cannot, therefore, be put down to preservation of the starting peptides alone: carrier digestion, peptide transformation and the release or generation of further sequences may all play a part.

Lipid vesicles offer a different delivery route, and their performance too is governed by the properties of the peptides. Mohan et al. [71] found that the molecular-weight range of whey peptides had little effect on overall encapsulation efficiency or nanoliposome size, but did affect how the peptides were distributed between the vesicle surface, the aqueous core and the lipid bilayer. In a later study, liposomes containing cationic whey peptides had a larger mean diameter, greater dispersity and slightly higher encapsulation efficiency than those containing anionic peptides, while peptide charge showed no clear effect on liposome surface charge or on peptide distribution within the vesicles. The authors accordingly drew a more cautious conclusion: that encapsulation efficiency and physicochemical properties may reflect the combined effects of peptide charge, compositional homogeneity and surface hydrophobicity [72]. Similar bulk encapsulation-efficiency values may thus conceal differences in peptide location, in peptide–phospholipid interactions and in subsequent release.

Corrêa et al. [21] encapsulated an antioxidant and ACE-inhibitory fraction from sheep-whey hydrolysate in phosphatidylcholine nanoliposomes. The vesicles averaged about 166 nm in diameter, remained physicochemically stable over 30 days, and retained roughly 87% of their initial antioxidant activity by the end of storage [21]. Mor et al. [22] prepared niosomes carrying low-molecular-weight casein peptides by high-shear homogenization; the vesicles had a mean diameter of 37.64 ± 0.98 nm, encapsulated about 67% of the peptides, retained their antioxidant activity, and released the peptides more slowly than the free peptides under simulated gastrointestinal conditions.

Gong et al. [74] more recently prepared casein-peptide liposomes with a mean diameter of 86.13 ± 0.62 nm and an encapsulation efficiency of 87.29 ± 0.82%. The formulation reduced peptide degradation and gave more controlled intestinal release during simulated digestion, and nutrient-absorption and microbiota-related outcomes were examined in experimental models rather than in human intervention [74]. Direct dairy-matrix evidence comes from Wang et al., who incorporated hyaluronic-acid-coated liposomal casein peptides into yogurt and followed the product for 21 days at 4 °C. The coated liposomes improved the retention of antioxidant activity and sustained peptide release during simulated digestion, while also altering water-holding capacity, syneresis, texture and instrumental sensory responses [75]. Carrier performance, this shows, must be assessed together with its effects on the food matrix, and not from peptide encapsulation and release measurements alone.

Between them, these studies demonstrate peptide loading, physicochemical stability, retained in vitro activity, controlled release and, in the work of Wang et al., compatibility with a yogurt matrix. They do not establish systemic peptide absorption, bioavailability in humans, or biological efficacy in vivo [21,22,74,75].

Release can also be controlled without vesicles. Giroux et al. [20] obtained encapsulation efficiencies above 93% for casein-derived peptides in water-in-oil-in-water double emulsions, but gastrointestinal release varied sharply with the degree of peptide hydrolysis and with the oil phase. Gastric release ranged from 4% to 42%; intestinal release exceeded 80% with digestible butter oil or linseed oil, yet stayed below 18% in emulsions made with indigestible mineral oil. High encapsulation efficiency did not predict release, which was governed chiefly by the composition of the oil phase, by digestive destabilization and by lipolysis.

Formulating the primary water-in-oil emulsion is itself a critical step in building such systems. Salum et al. [76] compared emulsifier type and concentration and the proportion of dispersed phase in casein-hydrolysate-loaded primary emulsions intended for subsequent conversion into double emulsions. PGPR-based formulations gave lower viscosity and smaller droplets than several alternative emulsifiers, remained stable for at least five days, and reached D90 values below 2 μm under the conditions selected [76]. That study dealt with the formation and short-term stability of the primary emulsion, not with peptide release during digestion or performance in a dairy food. Read together, Giroux et al. [20] and Salum et al. [76] show that emulsion formation, storage stability, encapsulation efficiency, and gastrointestinal release are separate performance attributes, none of which can be inferred from another.

Tavares et al. [77] produced β-lactoglobulin microparticles containing a bioactive peptide concentrate by heat-induced gelation followed by high-intensity ultrasound. Sonication reduced the mean particle size and gave relatively round, monodisperse particles at selected pH values, which supports their potential use as peptide carriers [77]. Particle formation and peptide retention are, all the same, an early technological demonstration rather than validation in a finished dairy product or an in vivo model. Sun et al. more recently incorporated the milk-derived peptide FDRPFL into a Pickering emulsion gel stabilized by whey- and soy-protein-isolate composite particles; the highest reported encapsulation efficiency was 72.8%, and the carrier improved peptide bioaccessibility during simulated digestion [78]. Gastrointestinal delivery is supported by these results; systemic bioavailability is not.

Milk-derived peptides may also serve as structural components of an emulsion rather than only as encapsulated cargo. Adjonu et al. [79] used whey-protein-derived peptide fractions as emulsifiers in oil-in-water nano-emulsions. The lower-molecular-weight fractions, with the stronger antioxidant and ACE-inhibitory activities, stabilized the interface relatively poorly, whereas the less bioactive >10 kDa fractions gave more stable nano-emulsions with droplet diameters of about 174–196 nm; stability also depended on peptide concentration, hydrolytic enzyme, storage time, and temperature. There is thus a potential trade-off between measured peptide bioactivity and the interfacial properties needed to form a stable food-delivery system.

Electrospun carriers give one of the clearer examples of peptide encapsulation followed by testing in a dairy matrix. Rajanna et al. [23] incorporated casein-derived peptides into pullulan nanofibers with mean fiber diameters of about 60–133 nm and encapsulation efficiencies of 72.95–86.04%. Roughly 75.3% of the peptides were released after 8 h under gastrointestinal-pH conditions, and the antioxidant activity of the released peptides was comparable to that of the unencapsulated material. Adding the nanofibers to milk produced no marked change in the physicochemical properties evaluated. The study demonstrates technological compatibility with a dairy matrix and release under controlled pH conditions; it does not establish peptide absorption, systemic bioavailability, or a health effect.

Evidence going beyond physicochemical characterization is still limited. Cian et al. [80] examined casein glycomacropeptide, its simulated gastrointestinal digest, and a microencapsulated preparation in two- and three-dimensional mouse jejunal organoid models. Digestion substantially changed the gene-expression effects of the glycomacropeptide, whereas microencapsulation largely preserved the effects seen in the three-dimensional model; in the two-dimensional model, almost no response was detected [80]. An outcome that depends on the model in this way shows why preserved chemical activity, bioaccessibility, and a reproducible biological effect belong at separate levels of evidence. The work remains an in vitro organoid study and offers no animal or human clinical evidence.

The clearest direct bridge between milk-peptide delivery and probiotic encapsulation comes from Soltani et al. [81] hydrolyzed whey protein, *Lactiplantibacillus plantarum,* and *Lacticaseibacillus casei* were incorporated into alginate beads coated with whey-protein isolate and added to a whey-permeate-based beverage. After 28 days of refrigerated storage, the coated formulation held higher probiotic counts and antioxidant capacity than the uncoated one; the coated beads gave about 7.68 log CFU/g after simulated gastrointestinal exposure and reduced the perceived bitterness of the hydrolysate [81]. Probiotic–peptide co-encapsulation in a dairy-derived beverage matrix is therefore demonstrated. The authors suggested that the whey-protein hydrolysate might support probiotic growth, but neither selective substrate utilization nor a health benefit was shown, and since the preparation contained no separately validated prebiotic substrate, it should not be classified as a confirmed three-component PRO–PRE–peptide system.

Taken as a whole, the available evidence shows that selected carriers can protect milk-derived peptide fractions, modify their release during simulated digestion, retain the activities measured in vitro, and be incorporated into milk, yogurt, or whey-based formulations. These outcomes depend on peptide composition, carrier architecture, processing conditions, and the food matrix, and none of them establishes any carrier as universally superior. Most studies stop at physicochemical characterization, storage experiments, simulated digestion or in vitro assays, and no fully validated dairy system combining a probiotic, a confirmed prebiotic substrate and an independently characterized milk-derived bioactive peptide in one delivery platform was identified. Peptide encapsulation is thus a complementary technological foundation for future integration with PRO–PRE systems, not evidence that validated three-component formulations already exist.

Future peptide-delivery studies should identify the relevant sequences before and after processing and digestion, determine whether activity survives release, and keep gastrointestinal bioaccessibility distinct from intestinal absorption and systemic bioavailability.

### 3.5. Applications in Dairy Products

This section deals with studies in which encapsulated systems were evaluated in an actual dairy or dairy-derived product. The outcomes at issue are viable-cell retention under specified storage conditions, survival in simulated gastrointestinal models, and changes in physicochemical, rheological, and sensory properties. Systems are classified by the architectural criteria defined in Section 2.5, so a product containing an encapsulated probiotic together with a separately added prebiotic is not treated as a confirmed PRO–PRE co-encapsulation system.

Dairy-matrix studies generally show that encapsulation reduces probiotic losses relative to free-cell controls, though how much protection is obtained varies with the strain, the carrier, and the product [11,13,49,82,83]. The same carrier may behave differently in liquid, concentrated, and frozen products, and incorporating a proposed prebiotic does not necessarily improve viability beyond what the carrier itself achieves [11,35,84]. The studies considered below also provide technological, storage, and simulated-digestion evidence rather than evidence of a health benefit in humans.

#### 3.5.1. Yogurt and Fermented Milk

Alginate–gelatin microgels containing *Lactobacillus acidophilus* are among the better-characterized examples of PRO–PRE application in yogurt. Silva et al. [49] compared probiotic microgels with and without fructooligosaccharides (FOS). Both carriers improved survival over free cells during yogurt storage and simulated gastrointestinal exposure, and the FOS gave a more interconnected carrier network and additional protection under the conditions tested.

Working with related alginate–gelatin systems, Cezarino et al. [11] compared the carrier in liquid, Greek and frozen yogurts. Viability during storage was highest in liquid yogurt, whereas the frozen formulation gave the strongest protection during simulated digestion. Up to 20% probiotic- or FOS-containing microgels left apparent viscosity unchanged in the liquid and frozen products but reduced it in Greek yogurt. Carrier performance and its effect on texture thus depend on the composition and physical structure of the yogurt matrix [11].

Other co-encapsulation studies gave mixed technological outcomes. Li et al. [85] incorporated *Lacticaseibacillus casei* 30-1 and lactitol into the same microcapsules; survival during storage improved, but the authors reported poorer yogurt-quality characteristics than in the controls. Ismail et al. [14] co-encapsulated *L. acidophilus* with xylo-oligosaccharides in an alginate–whey-protein-concentrate matrix and reported 10.45 log CFU/mL after 20 days in stirred yogurt. Both support the technological feasibility of putting co-encapsulated cultures into yogurt, but they cannot be compared directly, since the studies differed in strain, carrier composition, initial cell load, storage protocol, and quality measurements [14,85].

A useful negative result comes from Pinto et al. [35], working with Greek-style yogurt containing spray-dried *Bifidobacterium animalis* subsp. *lactis* BB-12. Probiotic counts held steady for 28 days in yogurt with free cells or with cells encapsulated in sweet whey, while a slight decrease occurred where inulin had been included in the microcapsules. After storage, the loss of viability during simulated gastrointestinal digestion was greater in the sweet-whey–inulin treatment than in some comparator formulations, and the microcapsules also changed selected physicochemical and textural properties of the yogurt. Inulin gave no additional protection in this particular formulation, and cannot be assumed to improve every probiotic carrier.

The distinction between co-encapsulation and separate incorporation matters particularly for fermented milk beverages. Hashemi et al. [15] studied Doogh containing alginate-encapsulated *Lactiplantibacillus plantarum* LS5, with *Helianthus tuberosus* inulin added separately to the beverage. Over 22 days of refrigerated storage, encapsulation reduced the loss of viable cells, while inulin improved the survival of both free and encapsulated cells. Neither treatment significantly changed the sensory attributes evaluated, although encapsulation affected exopolysaccharide production and phase separation [15]. This was a formulation with an encapsulated probiotic and an externally added prebiotic, not a confirmed co-encapsulation system.

In a more recent Doogh study, Ahmadi et al. [86] compared plain alginate carriers with alginate formulations containing yeast-cell-wall material or β-glucan. After 28 days at 4 °C, the alginate–yeast-cell-wall formulation retained more than 7 log CFU/mL of *Lactobacillus acidophilus* LA-5 and gave the strongest overall survival through sequential simulated gastric and intestinal exposure. The β-glucan formulation raised beverage viscosity without impairing sensory acceptance [86]. Since neither selective substrate utilization nor a health benefit was evaluated, these formulations should be regarded as co-localized candidate PRO–PRE architectures rather than validated synbiotics [8,86].

#### 3.5.2. Cheese

The most extensive cheese evidence comes from a related series of studies on white goat cheese, in which a probiotic culture was incorporated into extruded sodium-alginate capsules either alone or together with FOS or inulin. Over 180 days at 4 °C, the prebiotic-containing microcapsules generally maintained probiotic viability better than the corresponding free-cell treatments [82,87]. The capsules also modified cheese composition and ripening, with differences reported in dry matter, acidity, nitrogen fractions, organic acids, free amino acids and free fatty acids [10,88]. Sensory acceptance was not adversely affected under the conditions evaluated, though probiotic and prebiotic incorporation changed the volatile-compound profile during storage [89]. As these publications form a connected experimental series, they offer complementary analyses of one goat-cheese development program rather than independent replications [10,82,87,88,89].

Not every product described as synbiotic cheese held both components within the carrier. In the Gouda-type cheese of Borrás-Enríquez et al. [90], *Bifidobacterium animalis* subsp. *lactis* was spray-dried in reconstituted skim milk and a β-cyclodextrin–gum-Arabic matrix, while Nutriose^®^ FB was incorporated separately during cheese manufacture. Viable counts stayed above 8 log CFU/g through 40 days of ripening and above 7 log CFU/g after the simulated gastrointestinal procedure, with no significant change in the physicochemical variables examined [90]. What this demonstrates is the survival of an encapsulated probiotic in a prebiotic-containing cheese, not PRO–PRE co-encapsulation or a consumer health benefit [8,90].

Microencapsulation likewise improved the survival of *Lactobacillus acidophilus* LA-5 over 60 days at 4 ± 1 °C in ultrafiltered white cheese and during subsequent simulated digestion, but adding inulin gave no further advantage; counts remained at about 6 log CFU/g at the end of storage [84]. Here again, a prebiotic ingredient did not necessarily enhance the protection that encapsulation provides [35,84].

Cream cheese shows that better cell protection can coexist with measurable changes in the food matrix. De Andrade et al. [91] reported slightly higher counts for microencapsulated than for free *Lactiplantibacillus plantarum* CCMA 0359 after simulated digestion, 8.31 against 8.07 log CFU/g. Incorporating the probiotic, especially in encapsulated form, raised acidity and lowered pH during storage without significantly impairing consumer acceptance [91]. No prebiotic substrate was included, so the study supports the dairy compatibility of probiotic microcapsules but is not direct PRO–PRE evidence.

Bakhtiyari et al. [92] reported viability losses over 60 days at 4 °C of 3.19, 1.23, and 0.76 log CFU/mL for free *Lactiplantibacillus plantarum* combined with *Silybum marianum* seed extract, for encapsulated cells, and for cells co-encapsulated with the extract, respectively. Co-encapsulation also affected the antioxidant, physicochemical, and textural properties of the cheese [92]. The plant extract was not shown to be a selectively utilized prebiotic substrate, so the formulation is better regarded as a probiotic–bioactive co-encapsulation analogue than a confirmed PRO–PRE system [8,92].

#### 3.5.3. Ice Cream and Frozen Dairy Products

Maleki et al. [13] incorporated *Lacticaseibacillus rhamnosus* into ice cream in composite microcapsules of whey-protein isolate, bacterial cellulose, and inulin. Over 50 days of frozen storage, the decline was smaller for encapsulated than for free cells, 0.61 against 0.97 log CFU/g. This supports a protective effect under the formulation and storage conditions used, but the design does not identify which carrier component was mainly responsible.

Zanjani et al. [93] encapsulated *Lacticaseibacillus casei* and *Bifidobacterium adolescentis* in alginate matrices containing different starches and coated the particles with chitosan or poly-L-lysine. High-amylose corn starch gave the strongest survival over 100 days at −30 °C, and neither free nor encapsulated preparations significantly altered the sensory attributes or pH of the ice cream during storage [93]. The resistant-starch carrier is a candidate PRO–PRE architecture, but selective substrate utilization and a health benefit were not evaluated [8,93].

Hayayumi-Valdivia et al. [94] evaluated sodium-alginate microcapsules containing probiotic bacteria and mango-peel powder in ice cream stored for 180 days at −20 °C. The formulation with 2% mango-peel powder retained more than 6 log CFU/g at the end of storage and scored 7.6 for overall acceptability on the authors’ scale [94]. Mango-peel powder supplied a fiber-rich carrier component, but maintaining viable counts does not by itself demonstrate selective utilization of the substrate or establish its prebiotic status [8,94].

Taken together, the frozen-product studies show that encapsulation slows rather than prevents the loss of probiotic viability during storage [13,93,94]. They do not establish that inulin, resistant starch, or any other proposed prebiotic will consistently improve frozen-storage survival, since carrier composition, strain, freezing protocol, overrun, storage temperature, and storage duration were not standardized across the studies [13,93,94].

#### 3.5.4. Other Dairy Products

Whey-based beverages offer a way to combine probiotic delivery with the use of a dairy side stream. Saniani et al. [95] encapsulated *L. plantarum* in alginate microspheres containing inulin and dextran. The formulation with 1.5% inulin and 0.5 mg/L dextran gave the highest reported encapsulation efficiency, 93.55%, and protected the cells better than plain alginate during acidic and bile exposure. Beverages containing the microencapsulated cells held higher probiotic counts than those with free cells, though pH, acidity, and volatile composition also changed. The study gives direct evidence of a shared probiotic–inulin carrier in a whey beverage, but the encapsulation efficiency reported should not be equated with long-term survival or a health effect.

In chocolate milk, Ajlouni et al. [16] compared free and microencapsulated lactic acid bacteria with 2% or 4% konjac root powder added separately to the product. After 21 days, the encapsulated-cell treatments contained about 4.91 and 5.00 log CFU/mL, compared with 3.85 log CFU/mL in the control the authors identified, and konjac powder markedly increased viscosity, particularly early in storage. A growth-supporting effect within the food matrix is suggested, but the system was not a true PRO–PRE co-encapsulation architecture, since the konjac powder was incorporated separately from the encapsulated bacteria.

Das et al. [96] developed a non-fermented milk pudding containing beads in which a probiotic culture was co-encapsulated with ascorbic acid, with isabgol being incorporated separately as a proposed prebiotic ingredient. The optimized product contained 9.457 log CFU/g and 38.21 mg ascorbic acid per 100 g and received acceptable sensory scores. This is multicomponent co-delivery in a dairy product rather than co-encapsulation of the probiotic with the proposed prebiotic: the beads held the probiotic and the vitamin, while the isabgol sat in the surrounding product.

#### 3.5.5. Comparative Interpretation

The most consistent finding across the dairy applications is a smaller decline in viable counts for encapsulated cells than for the corresponding free-cell controls [11,13,49,82]. Evidence that putting a prebiotic into the same carrier adds protection is less consistent. Positive results were reported for selected FOS-, lactitol-, xylo-oligosaccharide- and inulin-containing systems, whereas sweet-whey–inulin microcapsules in Greek-style yogurt and inulin supplementation in ultrafiltered cheese did not improve performance [14,35,49,84,85].

The strength of the product evidence also varies by matrix. Yogurt studies give useful comparisons between carrier compositions and product structures, while showing that microgels may alter viscosity, texture, or overall quality [11,35,85]. Cheese allows relatively long storage and ripening assessments, though much of the direct goat-cheese evidence comes from a related experimental series and needs independent replication [10,82,87,88,89]. Ice-cream studies demonstrate probiotic retention through prolonged frozen storage, but differences in storage temperature, strain and carrier composition rule out any direct ranking of the proposed prebiotic materials [13,93,94]. For whey beverages, chocolate milk and non-fermented dairy desserts, the evidence is thinner still, resting on a small number of formulation studies [16,95,96].

Suitability for dairy use should therefore be judged on at least four separate outcomes: viable-cell retention relative to a free-cell control; stability over the intended storage time and temperature; survival and release in a clearly described digestion model; and effects on the physicochemical and sensory properties of the final product [10,11,13,35,82,87,88]. None of these on its own demonstrates selective substrate utilization, synbiotic functionality, or a health benefit, all of which require further evidence under the consensus definition [8]. The dairy studies available establish technological feasibility at product level; none of those reviewed in this section provides direct human clinical validation of the complete encapsulated formulation.

Table 2 summarizes the carrier architecture, storage conditions, viable-cell results, and product-quality effects reported in each study.

### 3.6. Regulatory, Commercial, and Scale-Up Considerations

Encapsulation that succeeds in the laboratory does not make a PRO–PRE dairy product commercially feasible. Translation depends on three things: the probiotic strain and all carrier constituents must be suitable for the intended food use; the manufacturing process must be reproducible at the scale required; and the product must hold its microbiological, physicochemical, and sensory specifications throughout its declared shelf life. The evidence needed to classify the product, and to support any health-related claim, is again something separate from measurements of encapsulation efficiency, particle morphology or survival in simulated gastrointestinal digestion.

Safety assessment begins at strain level. Current recommendations for micro-organisms intended for use as probiotics call for unequivocal taxonomic identification, preferably supported by whole-genome sequencing; assessment of the history and conditions of use; screening for relevant virulence determinants and for acquired antimicrobial-resistance genes that could be transferred; and verification that the strain is still viable at the evidence-based dose at the end of shelf life. Products intended for infants, older adults, immunocompromised individuals or other vulnerable populations require particular attention [97]. Safety status and probiotic status are not interchangeable: a microorganism may be considered suitable for food use while lacking the clinical evidence that would qualify it as a probiotic.

In the European Union, the Qualified Presumption of Safety framework pre-assesses biological agents generically, based on taxonomic identity, the body of knowledge available and any potential safety concerns. QPS status is not authorization of a particular product, and where qualifications or safety concerns apply, it does not replace strain- and use-specific assessment; for bacterial taxonomic units, absence of acquired resistance to therapeutically relevant antimicrobials remains an important qualification [98]. The position in the United States is different. A substance added intentionally to food generally requires food-additive authorization unless its intended use is generally recognized as safe or otherwise exempt, and a GRAS conclusion is specific to the substance, its intended conditions of use and the safety evidence available: it establishes no probiotic efficacy and substantiates no health claim [99]. Regulatory status must accordingly be established for the market intended, and not inferred from approval or use in another jurisdiction.

The carrier needs the same scrutiny as the strain. Prebiotic substrates and wall materials, whether proteins, polysaccharides or lipids, along with emulsifiers, crosslinkers, coatings and processing aids, must all comply with the conditions attached to their intended food use. Calling a material food-grade in a research paper settles nothing on its own, since authorization is specific to the application, the concentration and the manufacturing purpose; residues left by the process, microbiological purity, allergen declaration and compositional specification must be settled as well. In the European Union, only additives on the Union list may be used, under the conditions given there [100]. A delivery system made by a new process that significantly changes the composition or structure of the food may also come under the Novel Food Regulation, as may one meeting the legal definition of an engineered nanomaterial [101], and an ingredient that does meet that definition carries the word “nano” in brackets after its name in the ingredient list [102]. A laboratory measurement placing some particles in the nanometer range does not, by itself, put a carrier into that legal category.

How many viable cells a serving needs at consumption depends on the evidence for that strain and that benefit; there is no universal figure. The 10^6^–10^7^ CFU/g or mL quoted throughout the food literature is a technological convention rather than a regulatory requirement. Development work should fix a dose from the efficacy evidence and then show, with validated enumeration, that the dose is still present at the end of the declared shelf life under the storage conditions stated [97]. Encapsulation complicates the count itself: cells that remain inside an incompletely disrupted carrier will be recorded as dead unless the recovery step has been validated.

Claims are a separate matter again. A microorganism and a candidate substrate sharing one carrier, better survival in storage or in a digestion model, short-chain fatty acids produced in vitro: none of these on its own establishes a synbiotic health benefit. ISAPP asks for a demonstrated health benefit from the combination, and for a synergistic synbiotic, evidence that the co-administered microorganism utilizes the substrate selectively [8]. Satisfying the scientific criteria is still not the same as satisfying the rules of a given market. In the European Union, a health claim must be substantiated, authorized and listed in the applicable register before it is used in commercial communication [103], and Commission guidance has treated wording such as “contains probiotics” or “contains prebiotics” as potentially implying a health benefit, with enforcement and permitted wording varying between national markets [104]. Product names, labels and promotional material should therefore be checked against the target jurisdiction before commercialization.

Scale-up is best judged against critical quality attributes fixed in advance, rather than on the assumption that laboratory conditions will transfer as they stand. Strain identity and purity, viable-cell recovery and batch-to-batch variation belong on that list, as do particle-size distribution, moisture content and water activity, carrier composition, mechanical stability, release behavior, microbiological quality and compatibility with the dairy matrix intended. Pilot-scale trials then show whether altered mixing, pumping, shear, heat and mass transfer, residence time or drying rate change cell survival or carrier structure, and whether the downstream steps hold up: particle recovery, washing or drying, hygienic equipment design, cleaning, packaging, dependence on the cold chain and stability through real transport and storage. Sensory evaluation is also required because sedimentation, visible particles, grittiness, excessive viscosity, changes in flavor or peptide-associated bitterness may compromise the commercial acceptability of a carrier that otherwise performs well according to microbiological criteria [105,106,107].

Cost is best counted per acceptable serving delivering the required viable dose, encapsulation efficiency being silent on the economics. Biomass yield and fermentation medium, carrier ingredients, process losses, energy and water, drying or refrigeration, particle recovery, analytical quality control, packaging, storage and distribution all contribute. What matters is the sum across the chain, since improving survival at one stage may cost disproportionately at another. Archacka et al. [108] took this approach in probiotic production, treating culture-medium optimization and protective spray-drying formulations together rather than optimizing each operation on its own.

Few of the studies identified in this review went that far. Full techno-economic assessments and documented validations at commercial scale of dairy PRO–PRE co-encapsulation systems were uncommon; most used laboratory batches and reported short-term viability, particle characteristics or survival in simulated digestion, with comparatively little on long-term storage, manufacturing reproducibility, sensory acceptance, regulatory classification or cost per serving. Translation will therefore need pilot-scale process validation, shelf-life and distribution studies under realistic conditions, sensory testing of the actual product, safety assessment for the specific strain and formulation, and an early reading of the applicable regulatory route. Requirements of this kind say more about commercial readiness than broad market-size forecasts, whose figures depend heavily on how the encapsulation market has been defined.

Comparisons between studies are still limited by differences in probiotic strain, carrier composition, storage conditions, viable-cell recovery procedures and simulated gastrointestinal models. Findings from in vitro digestion should therefore be interpreted as formulation-specific technological evidence, not as proof of intestinal persistence or of a health benefit. Future studies should report initial and final viable counts, storage time and temperature, carrier architecture, component concentrations, particle-size distribution, validated recovery procedures and the corresponding free-cell controls. Wider use of harmonized digestion protocols, followed where appropriate by animal or human validation, would make the evidence both easier to compare and more useful for translation.

## 4. Future Directions

Inconsistent outcome definitions, heterogeneous storage and digestion protocols, and differences in microbiological recovery methods all limit how far the existing evidence can be compared, and future studies should begin by addressing them: through harmonized reporting and digestion protocols, and by evaluating complete formulations under realistic manufacturing and shelf-life conditions, with free-cell and probiotic-only carrier controls and, where relevant, a separately incorporated substrate control.

Synbiotic claims should be matched to the architecture proposed. Evidence of a health benefit is required for complementary and synergistic synbiotics alike; a claim of synergy requires in addition that the co-administered microorganism be shown to utilize the substrate selectively. Peptide-containing systems require more still: characterization of peptide identity and sequence-dependent stability, confirmation that biological activity survives processing, digestion and release, assessment of gastrointestinal bioaccessibility, and compatibility testing against the intended probiotic strain. Pilot-scale reproducibility, safety, regulatory status, sensory acceptance and cost should be established before commercial translation is attempted.

## 5. Conclusions

Across the dairy studies reviewed, encapsulation generally reduced probiotic losses during processing, storage, and simulated gastrointestinal digestion, though the degree of protection varied with the strain, the carrier, the encapsulation process, and the food matrix, and incorporating a proposed prebiotic into the same carrier was not consistently shown to add to it. No encapsulation method proved superior across all the outcomes evaluated. Bringing a probiotic and a proposed prebiotic into one carrier demonstrates physical co-localization, and no more: selective substrate utilization, functional synergy, and a synbiotic health benefit each must be demonstrated separately wherever they are claimed.

Studies of milk-derived peptide delivery reported retained in vitro activity and controlled or carrier-dependent release in selected systems, but no validated dairy platform combining a probiotic, a confirmed prebiotic and an independently characterized milk-derived bioactive peptide was identified. Most of the available evidence comes from laboratory-scale experiments, simulated-digestion models, or short-term product trials. Evidence of biological efficacy in vivo and of systemic bioavailability is limited, direct human clinical validation of complete co-encapsulated formulations is absent, and sensory performance and industrial feasibility remain insufficiently evaluated.

## Figures and Tables

**Figure 1:**
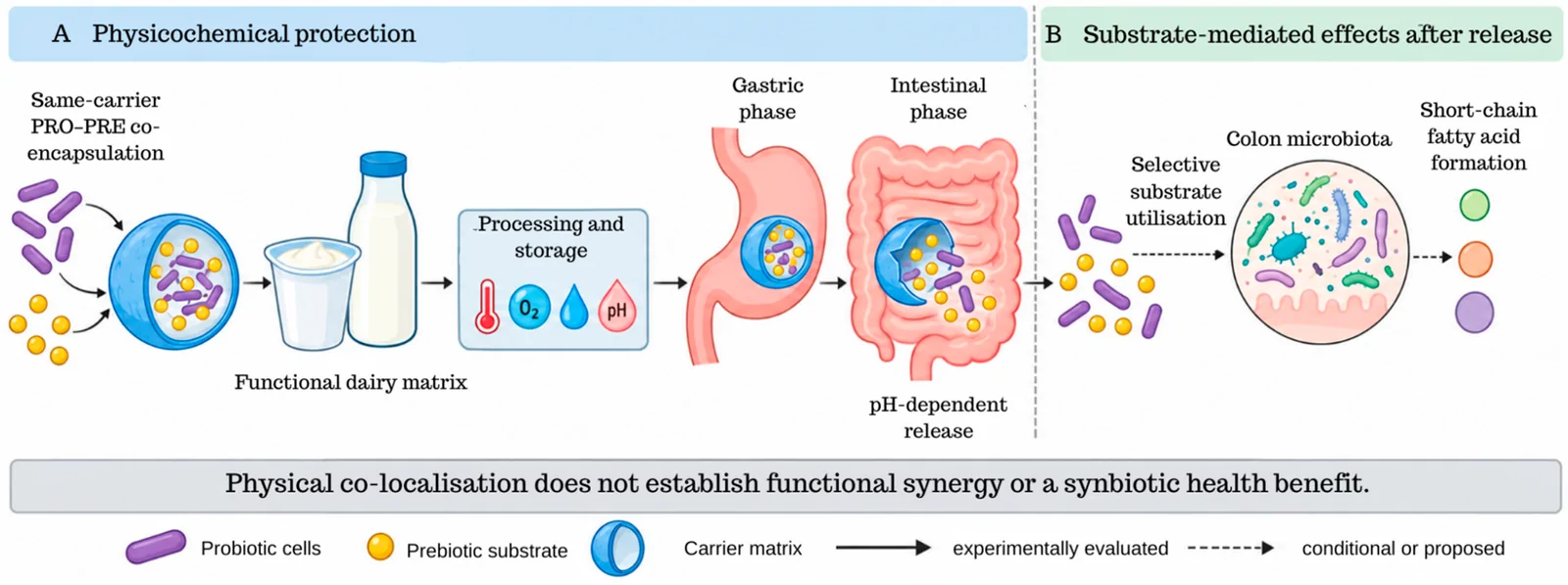
Conceptual representation of physicochemical protection and substrate-mediated effects in same-carrier probiotic–prebiotic (PRO–PRE) systems. Solid arrows indicate experimentally evaluated stages, whereas dashed arrows represent conditional or proposed effects. Selective substrate utilization and short-chain fatty-acid formation are strain-, substrate-, and model-dependent. Physical co-localization alone does not establish functional synergy or a synbiotic health benefit.

**Figure 2:**
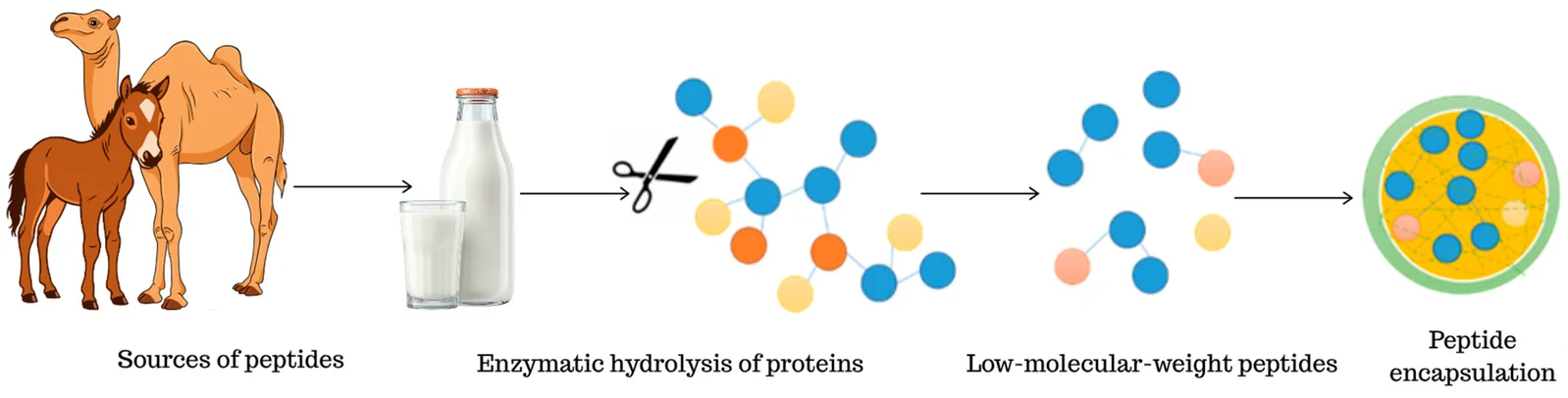
Production of milk-derived bioactive peptides and their encapsulation in protective delivery systems. Colors are used for schematic illustration only and do not indicate specific chemical identities.

**Table 2:** Selected dairy studies of encapsulated probiotics, proposed PRO–PRE systems, and related co-delivery architectures.

Probiotic and Carrier	Architecture	Dairy Product	Storage/Assessment	Main Viability Result	Product Effects and Limitation	Reference
*Lactobacillus acidophilus* in alginate–gelatin microgels, without or with FOS	Probiotic-only carrier vs. shared PRO–PRE carrier	Liquid, Greek and frozen yogurt	28 d; chilled or frozen storage; simulated GI digestion	Liquid yogurt gave the highest storage viability, whereas frozen yogurt provided the greatest protection during simulated digestion; quantitative endpoint values were not extracted from the accessible study record	Apparent viscosity was unchanged in liquid and frozen yogurt but decreased in Greek yogurt; sensory acceptance was not evaluated	[11]
BB-12 spray-dried in sweet whey, without or with inulin	Free cells vs. probiotic-only carrier vs. shared PRO–PRE carrier	Greek-style yogurt	28 d at 4 °C; simulated GI digestion after storage	Counts remained stable with free cells and sweet-whey capsules; the inulin formulation declined slightly and showed the greatest loss during digestion	Selected physicochemical and textural properties changed; no additional protection attributable to inulin was demonstrated	[35]
Alginate-encapsulated *Lactiplantibacillus plantarum* LS5; inulin added to the product	Encapsulated PRO + external PRE	Doogh	22 d at 4 ± 1 °C	Viability losses were approximately 2.70 log units for free cells and 1.71 log units for encapsulated cells; inulin improved survival in both groups	Capsules slowed phase separation; assessed sensory attributes were not significantly affected	[15]
Probiotic mixture in alginate capsules, without or with FOS or inulin	Probiotic-only vs. shared PRO–PRE carriers, with free-form comparators	White goat cheese	180 d at 4 ± 1 °C; simulated GI assessment in a related report	At day 180, encapsulated *Lacticaseibacillus paracasei* remained at 9.75 log CFU/g (10.36 initially), compared with 6.59 log CFU/g for free cells; FOS- and inulin-containing capsules gave higher counts than probiotic-only capsules. Exact *Bifidobacterium longum* endpoint values were not extracted	Volatile, organic-acid, amino-acid and fatty-acid profiles were affected; no adverse effect on assessed sensory attributes was reported	[10,82,87,88,89]
Spray-dried *Bifidobacterium animalis* subsp. *lactis* in skim-milk/β-cyclodextrin–gum-Arabic carriers; Nutriose^®^ FB added separately	Encapsulated PRO + external PRE	Gouda-type cheese	40-d ripening; simulated GI digestion after ripening	Counts remained above 8 log CFU/g during ripening and above 7 log CFU/g after digestion	Main physicochemical properties were not significantly affected; not a shared PRO–PRE carrier	[90]
*Lactiplantibacillus plantarum* and *Silybum marianum* seed extract in WPI-coated alginate beads	Probiotic–plant-extract co-encapsulation analogue	Cheese	60 d at 4 °C	Reported losses were 3.19 log units for free cells, 1.23 for encapsulated cells, and 0.76 for co-encapsulated cells	Antioxidant and textural properties changed; the extract was not validated as a prebiotic	[92]
*Lacticaseibacillus rhamnosus* and inulin in WPI–bacterial-cellulose composite capsules	Shared PRO–PRE carrier	Ice cream	50 d at −20 °C	Viability decreased by 0.97 log with free cells and 0.61 log with encapsulated cells	The composite-carrier design did not isolate the specific contribution of inulin	[13]
*Lacticaseibacillus casei* and *Bifidobacterium adolescentis* in alginate–starch capsules coated with chitosan or poly-L-lysine	Candidate shared carrier	Ice cream	100 d at −30 °C	High-amylose corn starch gave the highest survival after 100 d at −30 °C; quantitative endpoint values were not extracted reliably from the accessible primary record	No significant effect on pH or assessed sensory properties; selective utilization of starch was not tested	[93]
Freeze-dried mixed LAB in a whey-protein carrier; konjac root powder added separately	Encapsulated PRO + external proposed PRE	Chocolate milk	21 d at 4 °C	At day 21, counts were 4.91 and 5.00 log CFU/mL with 2% and 4% konjac powder, versus 3.85 log CFU/mL with encapsulated LAB but no konjac powder	Konjac powder increased viscosity; not a shared PRO–PRE carrier	[16]
*Lactiplantibacillus plantarum* in alginate–inulin–dextran microspheres	Shared candidate PRO–PRE carrier	Whey beverage	Beverage: 7 d at 4 °C; microspheres: 15 d at 4 °C; acid and bile tests	Reported EE was 93.55%; day-7 counts were 7.42 log CFU/g with encapsulated cells versus 5.75 log CFU/g with free cells	Physicochemical and volatile profiles changed; beverage storage was assessed for only 7 d	[95]

Note: PRO, probiotic; PRE, proposed prebiotic substrate; FOS, fructooligosaccharides; GI, gastrointestinal; EE, encapsulation efficiency; LAB, lactic acid bacteria; WPI, whey-protein isolate. “Shared PRO–PRE carrier” denotes physical incorporation of both components into the same carrier; it does not establish selective substrate utilization, functional synergy, or a synbiotic health benefit. Numerical results should not be compared directly across studies because strains, starting concentrations, enumeration procedures, storage conditions, and simulated gastrointestinal models differed. Values and units are reproduced as reported by the respective authors. Where numerical endpoint values could not be verified from the accessible primary record, this is stated explicitly rather than replacing them with a qualitative claim of improvement.

## Data Availability

No new data were created or analyzed in this study. Data sharing is not applicable to this article.
